# Effects of repeated cranial electrotherapy stimulation on physiological and behavioral responses to acute stress: a double-blind randomized clinical trial

**DOI:** 10.3389/fnhum.2025.1641801

**Published:** 2025-08-13

**Authors:** Kana Okano, Marissa Marko Lee, Hannah Hart-Pomerantz, Marisa Smith, Madelyn K. Sandone, Travis Harvey, Tad T. Brunyé

**Affiliations:** ^1^Center for Applied Brain and Cognitive Sciences, Tufts University, Medford, MA, United States; ^2^Teamworks Innovations Inc., Durham, NC, United States; ^3^U.S. Army DEVCOM Soldier Center, Natick, MA, United States

**Keywords:** clinical trial, stress, cranial electrotherapy stimulation, physiology, cognition, affect

## Abstract

**Background:**

Cranial electrotherapy stimulation (CES) is a low-intensity, pulsed neuromodulation technique widely marketed for reducing stress and anxiety. Despite its popularity, empirical evidence for its efficacy remains mixed, with few studies employing rigorous controls, standardized protocols, and repeated CES exposures.

**Objective:**

To evaluate whether repeated CES sessions can attenuate physiological, biochemical, cognitive, and affective responses to an acute laboratory stressor.

**Methods:**

A double-blind, randomized, placebo-controlled clinical trial was conducted with 46 healthy participants (27 military personnel, 19 civilians). Participants were randomized to receive either active CES (250–500 μA at 0.5Hz, individualized intensity) or sham stimulation for 20 sessions over approximately four weeks. At baseline and follow-up visits, participants underwent acute stress induction using torso shock; measures included physiological (heart rate, heart rate variability, respiration rate, pupil diameter), biochemical (salivary alpha-amylase, cortisol), cognitive (spatial orientation, recognition memory, decision-making), and affective (State-Trait Anxiety Inventory) indices.

**Results:**

Stress induction reliably elevated sympathetic-adrenal medulla (SAM) and hypothalamic-pituitary-adrenal (HPA) markers as well as subjective anxiety. However, across nearly all outcomes, active CES did not differ significantly from sham, nor were there interactions with session (baseline vs. follow-up). No meaningful group differences were observed in stress recovery, self-reported anxiety, or stress-related cognitive performance.

**Conclusions:**

These predominantly null findings challenge prevailing mechanistic accounts of CES and suggest limited efficacy in buffering acute stress responses in healthy, neurotypical individuals. Further controlled trials are needed to explore alternative parameters, populations, and neurophysiological endpoints to better understand CES’s therapeutic potential.

**Clinical trial registration:**

https://clinicaltrials.gov/, identifier NCT06034496.

## 1 Introduction

Cranial electrotherapy stimulation (CES) is a non-invasive transcranial electrical stimulation technique that delivers low-intensity pulsed alternating current through electrodes placed on the bilateral earlobes or temples (Feusner et al., 2012; Kirsch et al., 2014). CES has been applied to address a range of subclinical and clinical conditions, including anxiety, insomnia, and depression (Shekelle et al., 2018; Roh and So, 2017). To date, as many as 15 mechanistic models have been proposed to explain the neurophysiological effects of CES, yet no consensus has emerged (Liss and Liss, 1996; Brunyé et al., 2021). Criticisms, including lack of standardized protocols, double-blind designs, comprehensive outcomes, and robust statistics, have prompted calls for improved studies (Brunyé et al., 2021; Brunyé et al., 2022; Brunyé and Lee, 2025a,b; Kavirajan et al., 2014; Shekelle et al., 2018). Additional concerns about conflicts of interest and potential bias further underscore the need for more rigorous investigations (Price et al., 2021; Aseem and Hussain, 2019). In response, we conducted a double-blind, randomized, placebo-controlled clinical trial examining the effects of 20 repeated sessions of active versus sham CES on an array of outcome measures when participants are exposed to an acute stressor.

### 1.1 Cranial electrotherapy stimulation

Cranial electrotherapy stimulation emerged as a neuromodulation technique in the mid-20th century, initially developed in the Soviet Union as a non-invasive alternative to electroshock therapy (Brunyé and Lee, 2025a,b; Kerbikov, 1955; Wayne, 1960). Modern CES devices, such as the Alpha-Stim AID, are now portable and customizable, allowing tailored treatments for conditions like anxiety, depression, and sleep disorders (Price et al., 2021; Roh and So, 2017). CES devices are used increasingly in clinical and non-clinical settings, most employing bilateral earlobe electrodes to deliver currents to modulate central and/or peripheral nervous system activity (Feusner et al., 2012).

#### 1.1.1 CES, stress and anxiety

Anxiety disorders affect over 30% of the population, making them the most prevalent mental health disorders globally (Bandelow and Michaelis, 2015; Kessler et al., 2005). While conventional treatments like CBT and medication are effective, they face barriers such as cost, accessibility, and side effects (Mitte, 2005; Norton and Price, 2007). CES has shown moderate efficacy in modern studies, though methodological issues (Hearst et al., 1974; Iwanovsky and Dodge, 1968) and protocol variability limit strong conclusions regarding its promise as an adjunct to traditional treatments (Brunyé et al., 2021; Kavirajan et al., 2014; Shekelle et al., 2018).

Cranial electrotherapy stimulation has gained interest beyond clinical use for reducing stress and anxiety in healthy individuals, where even subclinical symptoms can impact wellbeing and behavior (Lazarus and Folkman, 1984; Cohen et al., 1983). As a non-invasive alternative to conventional therapies, CES may offer short-term relief without side effects or long-term commitments (Shekelle et al., 2018; Brunyé et al., 2021) with just 20–40 min of daily use (Price et al., 2020; Ching et al., 2022). Studies using stressors like dental procedures, public speaking, or cognitive tasks report reduced perceived stress, improved mood, and stabilization of heart rate variability (HRV) with active CES (Feusner et al., 2012; Barclay and Barclay, 2014), suggesting CES may help regulate the autonomic nervous system (ANS), shifting the autonomic balance toward increased parasympathetic activity and promoting relaxation (Howland, 2014; Asamoah et al., 2019).

Despite these findings, CES research in non-clinical populations remains limited. Many studies lack rigorous controls (Shekelle et al., 2018; Kavirajan et al., 2014) and show wide variability in stimulation protocols (Bikson et al., 2019; Santander et al., 2024; Paulus, 2011), making it hard to determine optimal use. While short-term benefits are reported (for conflicting evidence, see: Brunyé et al., 2022), long-term effects remain unclear (Brunyé et al., 2021; Ching et al., 2022).

#### 1.1.2 Neurophysiological mechanisms of CES

The neurophysiological mechanisms of CES remain debated, with over 15 proposed models categorized into macro-, meso-, and micro-level frameworks (Bestmann et al., 2015). At the macro level, CES may influence mood and behavior by modulating large-scale neural networks, such as the default mode network (DMN), which is involved in emotional regulation. Disrupting DMN hyperconnectivity may help reduce anxiety and depression (Feusner et al., 2012). CES may also increase alpha-band activity, linked to relaxation and decreased cortical excitability (Feusner et al., 2012; Kirsch et al., 2014). At the meso level, CES may enhance parasympathetic activity and reduce stress arousal by shifting autonomic balance, reflected in increased HRV (Roh and So, 2017; Howland, 2014). One possible mechanism is CES stimulation of vagal afferents, which influence brainstem autonomic centers. CES may also alter regional cerebral blood flow, particularly in the thalamus and brainstem, affecting arousal and sensory processing (Gense de Beaufort et al., 2012). At the micro-level, CES may influence neurotransmitter systems, increasing serotonin, dopamine, and norepinephrine to support mood and stress resilience (Liss and Liss, 1996; Roh and So, 2017). It may also reduce cortisol via hypothalamic-pituitary-adrenal (HPA) axis modulation, elevate GABA to calm neural circuits, and enhance acetylcholine activity for improved focus and emotional regulation.

While these models provide valuable insights, the lack of consensus highlights the need for rigorous studies to test these mechanisms. Importantly, Most CES studies focus on single-session effects, leaving it unclear whether repeated use yields cumulative benefits. Neuromodulation models suggest that ongoing CES may induce lasting changes in autonomic balance or neuroplasticity related to mood and stress regulation (Bestmann et al., 2015; Howland, 2014). By examining CES over 20 sessions, our study aims to determine whether repeated CES enhances resilience to acute stress. Understanding how CES affects stress and anxiety could improve its use and inform broader neurostimulation research.

### 1.2 The present study and hypotheses

To better understand the mechanisms underlying CES effects and its potential for managing stress in non-clinical populations, we conducted a comprehensive study examining physiological, biochemical, cognitive, and affective outcomes of CES administration.

Assessing biochemical markers of stress reactivity provides a gold-standard assessment of CES effects on the ANS. Herein, we examined markers of both SAM-related (via salivary alpha-amylase) and HPA-related (via cortisol) stress responses. These are our primary outcomes of interest given their quantitative foundation in the two phases of the acute stress response. Measuring physiological responses such as heart rate (HR), HRV, respiration rate (RR), and pupil diameter (PD) further allows us to understand how CES modulates autonomic and arousal states during stress exposure. We expect to find evidence for CES-induced decreases in salivary alpha-amylase (AA) and cortisol (CORT), increases in HRV, and/or decreased pupil dilation, HR, and/or RR during acute stress exposure, indicating parasympathetic activation and reduced stress reactivity in active (versus sham) CES conditions.

Affective measures, including subjective ratings of stress and anxiety, provide a complementary perspective by linking physiological and biochemical changes to individuals’ emotional experiences. If CES reduces tonic and/or phasic responses to stress, it should also lower subjective stress and anxiety ratings in the active vs. sham condition. Finally, cognitive assessments such as spatial cognition, recognition memory, and decision-making tasks were used to evaluate how CES influences stress-related performance decrements.

## 2 Methods

### 2.1 Participants and design

Fifty-eight healthy volunteers consented to this study (Table 1). Twelve withdrew with the final participant sample consisting of 46 volunteers (17 females, M_*age*_ = 22.3, SD_*age*_ = 4.8). Of these participants, 27 participants were military personnel (7 females, M_*age*_ = 21.8, SD_*age*_ = 5.1) and 19 participants were civilians (10 females, M_*age*_ = 23, SD_*age*_ = 4.4). The study was conducted in accordance with the ethical standards of the Declaration of Helsinki, and participants provided written informed consent in accordance with institutional review board (IRB) approvals from Tufts University and the United States Army (protocols 00003572 and 23-013, respectively). We used a double-blind, placebo-controlled design where participants were randomly assigned to the Active CES group or the Sham placebo group. The study was registered on ClinicalTrials.gov (NCT06034496) prior to data collection.

**TABLE 1:** Demographic characteristics of the two cranial electrotherapy stimulation (CES) groups (Active, Sham), and statistical comparison testing for baseline group differences across three demographic characteristics.

Demographic variable	CES group		Testing for baseline differences
	Active (M, SD)	Sham (M, SD)	Wilcoxon rank-sum test outcome
Age (years)	20.95 (2.94)	23.36 (5.82)	W = 216, *p* = 0.31
Education (years)	13.95 (1.43)	14.6 (3.04)	W = 264, *p* = 0.98
STAI-T	33.71 (10.75)	29.52 (7.28)	W = 328.5, *p* = 0.15
Sex	6 female, 15 male	11 female, 14 male	–
Handedness	20 right, 1 left	20 right, 5 left	–
Military or civilian	13 military, 8 civilian	15 military, 10 civilian	–
Race	7 white, 1 black, 10 Hispanic, 3 Asian, 0 other	16 white, 1 black, 3 Hispanic, 2 Asian, 3 other	–

### 2.2 Materials and equipment

For full details of the equipment used to collect data for this study, we refer interested readers to our previous (open access) publication (Brunyé et al., 2022). Briefly, CES was administered using the Alpha-Stim AID (Electromedical Products International, Inc., Mineral Wells, TX) device. The manufacturer preset and assigned private codes to each device, with intensity levels in 50 μA increments, from 250 to 500 μA, and designated each as either active or sham (no stimulation); intensities were independently verified by an electrical engineer. Devices and data were decoded to experimenters only at time of data analysis. Fully controllable active devices in which the intensity was able to be set from 50 to 500 μA were used for individualized intensity thresholding. The AID device was used with earclip electrodes, felt pads, and Alpha-Stim conducting solution.

Cardiorespiratory activity was measured using the Zephyr Bioharness 3 (Medtronic, Minneapolis, MN). Pupil dilation was tracked with SMI ETG2 eye-tracking glasses (SMI, Teltow, Germany). Stress was induced using the StressX Pro torso shock belt (Setcan, Winnipeg, MB, Canada). Saliva samples were collected via the Salimetrics Oral Swab method (Salimetrics, Carlsbad, CA), and AA and CORT were assayed by Hyperion Biotechnology (San Antonio, TX).

The recognition memory test (RMT) and spatial orienting test (SOT) were identical to those in Brunyé and Giles (2023). Briefly, the RMT involved learning a set of visual stimuli (i.e., people, vehicles, objects) to criterion and then being tested on recognition memory for those stimuli in virtual reality (VR). The SOT involved learning a map with labeled landmarks to criterion and then being tested on the ability to orient in the general direction of each landmark in VR. A decision-making test (DMT) was modeled after a traditional go/no-go task, where participants needed to distinguish people based upon what they were carrying. The task alternated between low and high workload periods, with three low-density blocks and three high-density blocks, in interleaved order. The software logged hits, misses, false alarms, and correct rejections, allowing us to calculate accuracy and discriminability (d’).

### 2.3 Virtual environment

Participants completed the RMT, SOT, and DMT in a large-scale VR system on an 8K-resolution (7,680 × 4,320) rear-projection screen (4.3 m × 2.4 m) occupying approximately 81° horizontal and 51° vertical field of view. Responses were wirelessly logged via handheld controllers.

### 2.4 Questionnaires and surveys

A demographics questionnaire collected information including age, sex, education, military experience, and firearm experience.

A daily questionnaire was developed to probe subjective physical experiences (e.g., headache, sleepiness, illness) on a four-point scale (anchored at None, Mild, Medium High).

A post-study questionnaire (blinding check) was developed asking participants whether they believed they had been in the Active or Sham condition.

Finally, the two subscales of the State-Trait Anxiety Inventory (STAI) (Spielberger, 1983) were used to measure situational anxiety (STAI-S) and trait anxiety (STAI-T).

### 2.5 Data collection procedures

Baseline Session (Day 1): Both Soldier and civilian participants provided written consent prior to their first session. All participants were seated in a private testing room, where they completed the demographics questionnaire and the daily questionnaire.

To account for individual differences in CES tolerance, a thresholding session was conducted to personalize CES intensities. Participants donned the earclip electrodes on both earlobes with the felt pads soaked in conducting solution, and received stimulation starting at 50 μA, increasing in 50 μA increments every 30 s. After each intensity, participants reported side effects using a four-level discomfort scale. Thresholding ended when a participant rated any symptom as Medium and linked it to CES, or when the maximum intensity (500 μA) was reached. A minimum threshold of 300 μA was required to participate. Once a threshold was established, a participant’s individualized CES intensity was set to 50 μA below their threshold (e.g., a 400 μA threshold resulted in 350 μA stimulation) to minimize side effects during their 20 CES sessions. If no symptoms were rated Medium or higher, and the participant reached the maximum intensity, they received 500 μA during their CES sessions.

**FIGURE 1:**
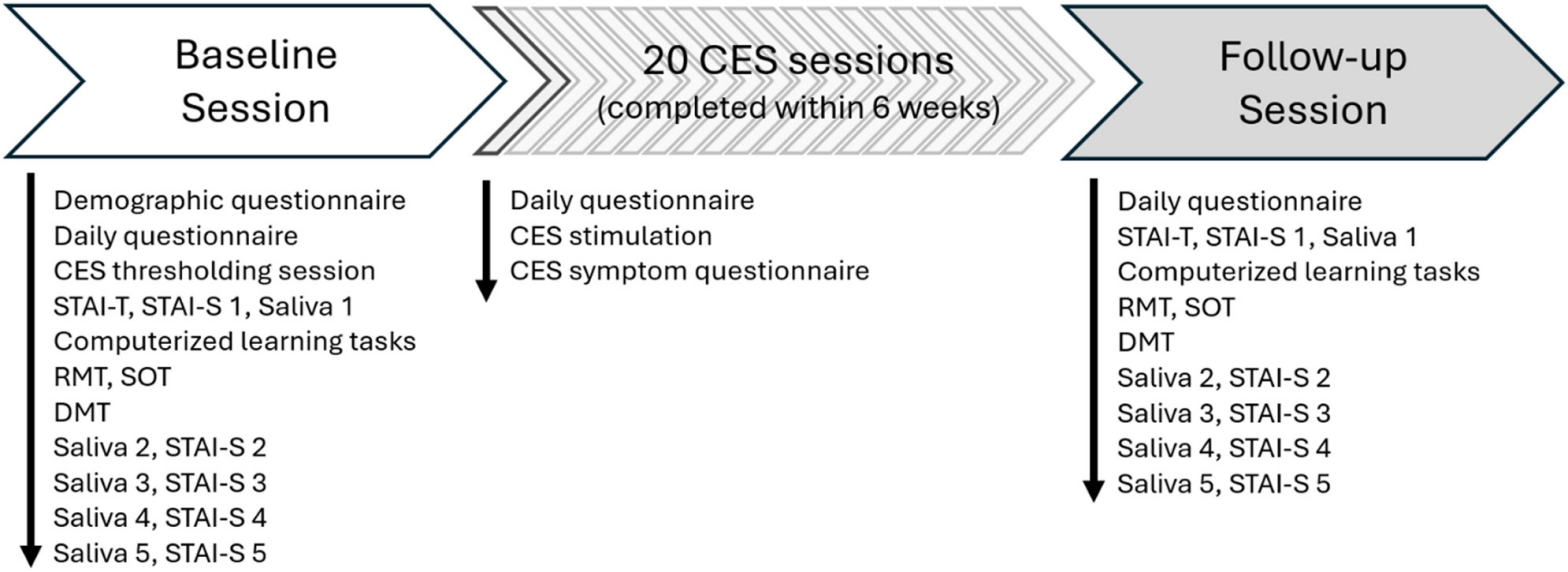
Timeline of study activities during the baseline session, 20 cranial electrotherapy stimulation (CES) sessions, and follow-up session.

Participants who reached threshold completed additional assessments, including the STAI-T, STAI-S, and a saliva sample. Participants then proceeded to a series of computerized learning tasks for the RMT, SOT, and DMT. All learning was done to a minimum accuracy criterion of 80%. Participants were then brought to the VR system where they donned the bioharness, eye-tracking glasses, and shock belt set to Level 3 intensity. Level 3 shock intensity is sufficient to elicit both SAM- and HPA-related responses (Brunyé et al., 2025) while lower levels are not (Brunyé et al., 2022). Participants then began the battery of cognitive tasks performed under the threat of torso shock for incorrect responses; the full timeline of study activities during the Baseline and Follow-up sessions is included in Figure 1. There were a total of five saliva samples and STAI-S responses, hereafter referred to as time points 1, 2, 3, 4, and 5.

#### 2.5.1 CES sessions (days 2–21)

For each of the 20 CES sessions, participants first completed the daily questionnaire. If participants reported any sickness, their session was rescheduled for a later date. If not, participants in the Active group received 20 min of active CES at their individualized intensity while participants in the Sham group received 20 min of inactive CES. Participants were instructed to relax, but not sleep during the session. Following the CES, they completed the post-stimulation side effects questionnaire. CES sessions took place at least 1 day apart from each other between the hours of 0800 and 1,700 (but not within 2 h of intended sleep onset), and participants had up to 6 weeks to complete all 20 CES sessions.

#### 2.5.2 Follow-up session (day 22)

The follow-up session mirrored the Baseline session with a few key differences: participants skipped the demographics questionnaire and CES thresholding. Participants also learned new people and objects for the RMT, new landmarks for the SOT, and new non-firearm (friendly) objects for the DMT.

### 2.6 Data processing and analysis

Baseline group differences were analyzed using Wilcoxon rank sum tests; Blinding Check was analyzed using binomial tests. All other analyses were conducted using mixed analysis of variance (ANOVA). Mixed ANOVAs were conducted using package rstatix (ver. 0.7.0; Kassambara, 2023) in R (ver 4.1.2). Full details on data processing and analysis can be found in the Supplementary materials.

## 3 Results

We collected complete CORT and survey data from 46 participants, AA data from 45 participants, RMT and SOT data from 40 participants, DMT and bioharness data from 37 participants, and pupil data from 28 participants. Statistical analyses were adjusted accordingly, as reflected in degrees of freedom.

### 3.1 Testing for baseline group differences

The Wilcoxon rank sum tests demonstrated that STAI-T scores, age, and education level of participants did not differ significantly as a function of CES Group (Table 1).

### 3.2 Immediate affective CES effects

The ANOVA showed no significant main or interactive effects of CES, Session, or Time (all *p’s* > 0.10), suggesting no immediate effect of CES administration on subjective anxiety during the 20 CES sessions.

### 3.3 Blinding check

In the Active group, 15 out of 21 participants (71.4%) guessed their CES group correctly, which was significantly above chance, *p* = 0.04. See Section 3.7 for a follow-up analysis of this pattern. In the Sham group, 17 out of 25 participants (68%) guessed correctly, which was not significantly different from chance, *p* = 0.05.

### 3.4 Manipulation check

For AA, HR, CORT, and STAI-S scores, independent samples *t*-tests demonstrated a successful stress induction with significant effects (i.e., pre- versus during-task) across all outcome measures (Table 2). Specifically, measures of SNS activity (AA, HR) increased (and RR, but not significantly), the measure of HPA axis activity (CORT) increased, and subjective anxiety assessments increased from pre-task to during-task (HR) or immediately post-task (AA, CORT, STAI-S).

**TABLE 2:** Manipulation check outcomes for alpha-amylase (AA), heart rate (HR), cortisol (CORT), and situational anxiety (STAI-S) scores, including mean differences and the results of independent samples *t*-tests, with effect size in Cohen’s D.

Outcome measure	Mean difference	*t*-test result
Alpha amylase	39.1 U/mL	*t*(44) = 3.01, *p* < 0.01, Cohen’s D = 1.54
Heart rate	19.84 BPM	*t*(34) = 9.09, *p* < 0.001, Cohen’s D = 0.44
Respiration rate	0.61 BPM	*t*(36) = 1.13, *p* = 0.27, Cohen’s D = 0.19
Cortisol	0.28 μg/dL	*t*(45) = 3.39, *p* < 0.01, Cohen’s D = 0.5
STAI-S	8.52 points	*t*(45) = 6.32, *p* < 0.001, Cohen’s D = 0.93

### 3.5 Physiological and biochemical responses to CES

All statistical results from physiological and biochemical responses to CES are detailed in Table 3. The ANOVA on HR showed a main effect of CES Group, with generally lower mean HR in the Active versus Sham group. There was no effect of Session. Critically, there was no interaction between CES Group and Session, suggesting a group difference that existed at Baseline and was not specific to a cumulative effect of CES.

**TABLE 3:** Statistical output of mixed analysis of variances (ANOVAs) assessing physiological and biochemical outcomes.

Effect	DFn	DFd	*F*	*P*	ηp^2^
**Heart rate**					
CES	1	35	6.00	0.01*	0.15
Session	1	35	0.98	0.33	0.03
CES: session	1	35	0.04	0.85	0.00
**Heart rate variability**					
CES	1	35	1.30	0.26	0.04
Session	1	35	1.93	0.17	0.05
CES: session	1	35	0.13	0.73	0.00
**Respiration rate**					
CES	1	35	0.27	0.60	0.00
Session	1	35	0.02	0.90	0.00
CES: SESSION	1	35	4.17	0.05	0.11
**Tonic pupil diameter**					
CES	1	26	0.70	0.41	0.03
Session	1	26	2.02	0.17	0.07
CES: session	1	26	0.31	0.58	0.01
**Alpha amylase**					
CES	1	43	2.7	0.10	0.06
Time	3.33	143	15.03	0.00***	0.26
Session	1	43	0.06	0.81	0.00
CES: time	3.33	143	1.01	0.40	0.02
CES: session	1	43	0.91	0.35	0.02
Time: session	4	172	4.88	0.00***	0.10
CES: time: session	4	172	1.02	0.40	0.02
**Cortisol**					
CES	1	44	1.82	0.18	0.04
Time	2.18	95.82	21.88	0.00***	0.33
Session	1	44	0.41	0.53	0.01
CES: time	2.18	95.82	2.73	0.07	0.06
CES: session	1	44	0.56	0.46	0.01
Time: session	2.93	128.88	3.90	0.01*	0.08
CES: time: session	2.93	128.88	1.26	0.29	0.03

*p < 0.05, ***p < 0.001.

The ANOVA on RR showed no main effects or interactions.

**FIGURE 2:**
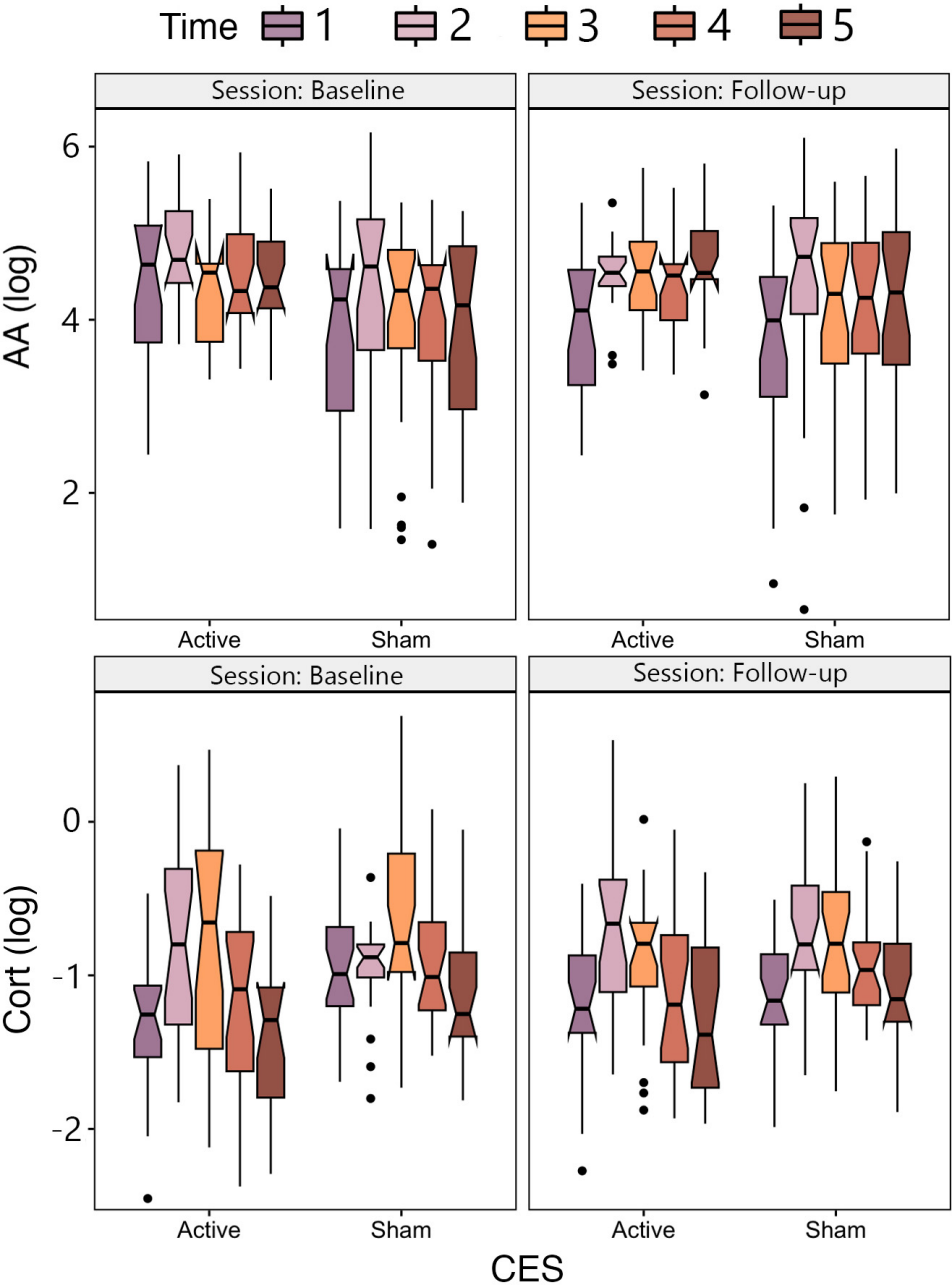
Notched box plots depicting mean AA **(upper panel)** and CORT **(lower panel)** as a function of CES group (Active, Sham) and session (Baseline, Follow-up).

The ANOVA on AA showed a main effect of Time, qualified by an interaction between Session and Time. For the interaction, AA levels tended to increase immediately after task performance and then decline over the next 60 min, and this effect was most pronounced in the Follow-up session. There was no main effect of CES Group or Session, or any interactions with CES Group (Figure 2).

The ANOVA on CORT showed a main effect of Time, qualified by an interaction between Session and Time. For the interaction, CORT levels tended to increase immediately after task performance and then decline over the next 60 min, and this effect was most pronounced in the Follow-up session. There was no main effect of CES Group or Session, or any interactions with CES Group (Figure 2).

The ANOVAs on HRV and tonic PD showed no main effects or interaction between CES Group and Session.

### 3.6 Cognitive and affective responses to CES

All statistical results from cognitive and affective responses to CES are detailed in Table 4.

**TABLE 4:** Statistical output of mixed analysis of variances (ANOVAs) assessing cognitive and affective outcomes.

Measure and effect	DFn	DFd	*F*	*P*	ηp^2^
**RMT accuracy**					
CES	1	36	0.04	0.85	0.00
Session	1	36	0.17	0.69	0.01
CES: session	1	36	0.73	0.40	0.02
**SOT direction error**					
CES	1	38	2.44	0.13	0.06
Session	1	38	1.31	0.26	0.03
CES: session	1	38	0.60	0.44	0.02
**SOT distance error**					
CES	1	38	6.65	0.01*	0.15
Session	1	38	1.50	0.23	0.04
CES: session	1	38	0.28	0.60	0.01
**DMT discriminability**					
CES	1	35	0.86	0.36	0.02
Session	1	35	20.02	0.00***	0.04
CES: session	1	35	0.002	0.96	0.00
**STAI-S**					
CES	1.00	44.00	0.72	0.40	0.02
Time	2.16	94.90	25.09	0.00***	0.36
Session	1.00	44.00	13.95	0.00***	0.24
CES: time	2.16	9490	1.61	0.20	0.04
CES: session	1.00	44.00	0.75	0.39	0.02
Time: session	2.19	96.25	4.88	0.00**	0.10
CES: time: session	2.19	96.25	0.99	0.38	0.02

*p < 0.05, **p < 0.01, ***p < 0.001.

The ANOVA on SOT distance error showed a main effect of CES Group, with lower overall SOT distance error in the Active CES group compared to Sham. There was no main effect of Session and no interaction between CES Group and Session.

The ANOVA on DMT discriminability showed a main effect of Session, with overall higher discriminability at Follow-up relative to Baseline. There was no main effect of CES Group or interaction between CES Group and Session.

The ANOVA on STAI-S scores showed main effects of Session and Time, qualified by an interaction between Session and Time. Overall, STAI-S scores tended to increase immediately following task performance then decline over the next 60 min. While the main effect of Time was significant in both Sessions (when tested in separate simple effects ANOVAs), the effect more robust during the Baseline session compared to Follow-up when comparing effect sizes (baseline ηp^2^ = 0.39, follow-up ηp^2^ = 0.17). There were no main or interactive effects of CES Group.

The ANOVAs on RMT accuracy, SOT direction error, and DMT response criterion showed no main effects or interactions.

### 3.7 Testing for placebo effects

Despite following the manufacturer’s guidelines for individualized thresholding of CES intensity, and similar techniques being used in prior research, we found that participants were able to correctly guess their CES condition at a level above chance. To test whether a placebo effect might drive CES effects, we reconducted our main analyses with participants divided into CES groups based on their *belief* of whether they received active versus sham CES. There were no main or interactive effects of CES in any of these analyses, demonstrating that any potential placebo effect due to lack of successful blinding was not robust enough to modulate any of our outcome measures.

## 4 Discussion

The study’s aim was to investigate whether 20 personalized CES sessions would mitigate physiological, biochemical, cognitive, and affective responses of acute stress. Our stress induction was effective, with measures of HR, subjective anxiety (STAI-S), AA, and CORT reliably increasing in response to the acute stressor.

We made three primary hypotheses about how CES would modulate responses to stress exposure. We did not support our first hypothesis, with no significant effects of Active versus Sham CES in HRV, PD, AA, or CORT. Though Active CES was associated with overall lower HR, the lack of interaction with Session suggests this pattern was present at both Baseline and Follow-up and thus cannot be attributed to CES. For our second hypothesis, while we replicated prior research showing robust stress induction as evidenced by significant pre- to post-stressor increases in subjective anxiety (Brunyé et al., 2025), this pattern was not modulated by Active versus Sham CES. For our third hypothesis, aside from a main effect showing lower distance-estimation errors in the SOT for the Active group, there was no Session interaction to suggest cumulative benefits of repeated CES. Both groups improved on the DMT from Baseline to Follow-up, mirroring practice effects without a differential CES contribution. Overall, findings offered minimal evidence that repeated active CES conferred advantages over sham.

### 4.1 Theoretical implications

Our mixed evidence raises important questions regarding the many proposed mechanisms underlying CES effects on neurophysiology, behavior, and affect. First, we did not observe the expected group-by-session interactions when examining salivary AA or CORT, which are key biomarkers of SAM- and HPA-axis activity, respectively (Sapolsky et al., 2000; Nater and Rohleder, 2009). This result challenges the HPA-attenuation model and questions whether 20 repeated CES sessions are sufficient to re-calibrate stress-related endocrine pathways. Second, despite some hints of a lower overall HR in the Active group, we did not detect a robust parasympathetic shift in other autonomic measures, such as HRV and RR, which are often cited as central to autonomic-based models of CES (Howland, 2014; Brunyé et al., 2021). Although the Active group’s lower overall HR could be viewed as partial evidence for a mild parasympathetic effect, the lack of a Session interaction indicates no evidence of cumulative or progressively beneficial effect of CES from Baseline to Follow-up. Finally, we found no cognitive advantages of Active CES at Follow-up relative to Baseline, making it difficult to reconcile our findings with micro-level models suggesting enhanced serotonin, norepinephrine, or GABA release following repeated CES (Liss and Liss, 1996; Aseem and Hussain, 2019), which on the surface may predict positive effects on cognitive function.

Taken together, our results neither robustly support nor decisively refute any single mechanistic model. They do, however, emphasize calls in the literature for more precise mechanistic investigations that combine high-resolution neuroimaging, well-powered designs, and carefully documented stimulation protocols (Bikson et al., 2019; Brunyé et al., 2021; Shekelle et al., 2018).

### 4.2 Applied implications

A central motivation for studying CES in a non-clinical population concerns its potential for stress management and performance sustainment in demanding applied contexts (Brunyé et al., 2021). Chronic or cumulative occupational stress can impair cognitive function, slow recovery from high-stress experiences, and elevate the risk of stress-related disorders (Bandelow and Michaelis, 2015). CES offers a portable, non-invasive, and relatively low-burden solutions for maintaining readiness without notable side effects (Price et al., 2021; Roh and So, 2017).

Contrary to some prior reports suggesting CES confers benefits in mitigating stress or promoting recovery (Barclay and Barclay, 2014; Kirsch and Nichols, 2013), the present study failed to document evidence of reduced physiological reactivity in our Active CES group. The practical implication is that 20 CES sessions, implemented under the manufacturer’s recommended procedures did not robustly diminish stress responses or protect against acute stress-induced performance decrements. Future research could explore alternative intensities, session schedules, or device parameters that might produce more reliable or cumulative effects

### 4.3 Limitations and future directions

Strengths of our clinical trial include a robust double-blind, randomized and placebo-controlled design, a larger sample size than most prior CES research, personalized and repeated CES administration, a comprehensive battery of outcome measures including physiological, biochemical, cognitive, and affective measures, a highly successful stress induction technique, detailed and transparent methods and statistical procedures, and no conflicts of interest among study team members. However, there are a few methodological and practical constraints that limit the generalizability of our findings and warrant continuing research.

First, although our sample size was comparable to or larger than prior CES trials (Brunyé et al., 2021; Ching et al., 2022; De Felice, 1997; Kavirajan et al., 2014), the sampling of both military personnel and civilians may have introduced heterogeneity in prior stress exposures and physiological baselines and reactivities.

Second, while we followed standard blinding procedures, a higher-than-chance proportion of Active CES participants correctly guessed their condition, which may have introduced unintentional expectancy effects. This was not simply a response bias, as many Sham CES participants also tended to correctly guess their condition. A follow-up statistical test grouping participants by the condition they believed they were in, showed no compelling evidence for a placebo effect across any outcome measures. Despite the double-blind design, improved sham methods, such as low-intensity stimulation that mimics cutaneous sensations, may better preserve blinding by reducing perceptible differences between groups (Ambrus et al., 2010; Bikson et al., 2018; Brunyé et al., 2014).

Third, although 20 sessions of CES is consistent with prior studies that found positive effects (Barclay and Barclay, 2014; Shekelle et al., 2018; Tan et al., 2011), it is unknown whether different stimulation intensities, session durations, or total number of sessions might yield stronger or more cumulative CES effects.

## 5 Conclusion

Overall, there was no strong support for the hypotheses that repeated CES decreases physiological or biochemical stress reactivity, subjective anxiety, or stress-induced cognitive performance decrements in healthy, neurotypical populations. The absence of definitive benefits in our data does not preclude the possibility that CES might help certain subpopulations, such as those with clinical or otherwise altered baseline stress or anxiety. Although the present study falls short of demonstrating clear advantages of CES in healthy, neurotypical populations, it offers a rigorous and transparent contribution to the growing literature on CES, one that can inform future targeted efforts to harness non-invasive neuromodulation for stress resilience and recovery.

## Data Availability

The datasets presented in this article are not readily available because, the datasets analyzed for this study are not available for public release due to potentially identifiable information in a relatively vulnerable military population. Requests to access the datasets should be directed to TB, thaddeus.t.brunye.civ@army.mil.
