# Supplementary material for: Effects of repeated cranial electrotherapy stimulation on physiological and behavioral responses to acute stress: a double-blind randomized clinical trial
Source: Front Hum Neurosci. 2025 Aug 13;19:1641801. doi: 10.3389/fnhum.2025.1641801 (PMC12380913; doi:10.3389/fnhum.2025.1641801)
Supplement: Supplementary file 1 [file Data_Sheet_1.docx]

**Supplementary Materials**

**Data Processing & Analysis**

*Baseline Group Differences.* To ensure Active versus Sham CES groups were similar at baseline, we compared their STAI-T scores, age, and education level. Because data violated assumptions of normality, we used Wilcoxon rank sum tests.

*Immediate Affective CES Effects.* To test for immediate affective effects of CES administration during the CES sessions, we conducted a mixed analysis of variance (ANOVA) to assess the effect of CES Group (2: Active, Sham), Session (1-20), and Time (2: pre-CES, post-CES) on STAI-S responses.

*Blinding Check .* To examine whether participants were able to correctly guess their assigned group (Active, Sham) above chance level (50%), we conducted binomial tests.

*Manipulation Check.* To ensure effective stress induction, we assessed AA, HR, CORT, and subjective stress responses to stress exposure during the baseline session. We compared sampled AA levels from immediately prior to (pre) to immediately after stress exposure (i.e., time points pre and 0-min post-task). We compared mean HR from immediately prior to (pre) to during stress exposure (i.e., during the RMT, SOT, DMT). We compared sampled CORT levels from immediately prior to (pre) to 0-minutes after stress exposure (i.e., time points pre and 0-min post-task). We compared STAI-S scores from immediately prior to (pre) to immediately after stress exposure. For these analyses, we conducted paired t-tests.

*Physiological & Biochemical Responses to CES.* To evaluate physiological and biochemical responses to CES, we used ANOVAs to assess the effect of CES Group (2: Active, Sham), Session (2: Baseline, Follow-up), and Time (pre-task, 0-min, 20-min, 40-min, 60-min post-task) on each of six outcome measures: HR, HRV, RR, tonic PD, AA, and CORT (number of levels varies as a function of outcome measure and is described as follows). Each response measure was assessed in a discretized time series based on its sampling times; continuous measures (e.g., HR) were discretized into pre-stress versus during-stress time periods. For HR, HRV, respiration, and tonic PD, pre-stress was calculated as the mean of the one-minute time period immediately preceding the beginning of the tasks, and during-stress was calculated as the mean of the entire behavioral task performance. For AA and CORT, we included all five time points in the analysis (i.e., pre-task, 0-min, 20-min, 40-min, 60-min post-task).

*Cognitive & Affective Responses to CES.* To evaluate the cognitive and affective responses to CES, we used mixed ANOVAs to assess the effect of CES Group (2: Active, Sham) and Session (2: Baseline, Follow-up) on each of four cognitive outcome measures, and one affective outcome measure. Cognitive measures included RMT overall accuracy, SOT absolute angular direction error, SOT absolute distance estimate error, DMT discriminability (d’), and DMT response criterion (c). Affective measures included STAI-S scores, and we used a mixed ANOVA to assess the effect of CES Group (2: Active, Sham), Session (2: Baseline, Follow-up), and Time (pre-task, 0-min, 20-min, 40-min, 60-min post-task).

Mixed ANOVAs were conducted using package rstatix (ver. 0.7.0; Kassambara, 2023) in R (ver. 4.1.2). Mixed ANOVA assumptions were checked using package rstatix (ver. 0.7.0; Kassambara, 2023) in R (ver. 4.1.2). Extreme outliers were identified, removed, and mean imputed. Normality was assessed with Shapiro-Wilk Normality Test; non-normal data was log-transformed. Homogeneity of variance was assessed with Levene’s Test and confirmed all groups had the same or similar variance. Mauchly’s test of sphericity was automatically conducted and corrected for in the mixed ANOVAs analysis.

*Exploring Intensity Covariate.* Based on reviewer feedback, we reconducted our main analyses with the inclusion of a covariate indicating stimulation intensity. The results of those analyses are found in Table S1; the inclusion of the covariate did not modify any of our primary results in a meaningful or consistent way. We also detail the number of participants that were assigned to each stimulation intensity level in Table S2. **Table S1.** Full ANOVA results for all outcome variables with individual-threshold CES *Intensity* included as a covariate.

| Outcome | Effect | DFn | DFd | F | *p* | *p* < .05 |
| --- | --- | --- | --- | --- | --- | --- |
| SOT Distance | Intensity | 1 | 37 | 0.277 | 0.602 |  |
|  | CES | 1 | 37 | 5.577 | 0.024 | * |
|  | Session | 1 | 37 | 0.348 | 0.559 |  |
|  | Intensity:Session | 1 | 37 | 0.09 | 0.765 |  |
|  | CES:Session | 1 | 37 | 0.192 | 0.664 |  |
| SOT Direction | Intensity | 1 | 37 | 0.337 | 0.565 |  |
|  | CES | 1 | 37 | 1.881 | 0.178 |  |
|  | Session | 1 | 37 | 0.488 | 0.489 |  |
|  | Intensity:Session | 1 | 37 | 1.022 | 0.319 |  |
|  | CES:Session | 1 | 37 | 0.979 | 0.329 |  |
| DMT d' | Intensity | 1 | 34 | 1.513 | 0.227 |  |
|  | CES | 1 | 34 | 1.37 | 0.25 |  |
|  | Session | 1 | 34 | 0.044 | 0.836 |  |
|  | Intensity:Session | 1 | 34 | 0.856 | 0.361 |  |
|  | CES:Session | 1 | 34 | 0.057 | 0.812 |  |
| DMT Response Criterion | Intensity | 1 | 34 | 0.097 | 0.757 |  |
|  | CES | 1 | 34 | 0.031 | 0.861 |  |
|  | Session | 1 | 34 | 1.322 | 0.258 |  |
|  | Intensity:Session | 1 | 34 | 1.414 | 0.243 |  |
|  | CES:Session | 1 | 34 | 1.063 | 0.31 |  |
| RMT Accuracy | Intensity | 1 | 35 | 0.306 | 0.584 |  |
|  | CES | 1 | 35 | 0.003 | 0.955 |  |
|  | Session | 1 | 35 | 0.115 | 0.737 |  |
|  | Intensity:Session | 1 | 35 | 0.062 | 0.805 |  |
|  | CES:Session | 1 | 35 | 0.577 | 0.453 |  |
| STAI-S | Intensity | 1 | 43 | 2.305 | 0.136 |  |
|  | CES | 1 | 43 | 1.289 | 0.262 |  |
|  | Time | 2.14 | 92.17 | 1.566 | 0.213 |  |
|  | Session | 1 | 43 | 3.203 | 0.081 |  |
|  | Intensity:Time | 2.14 | 92.17 | 0.166 | 0.861 |  |
|  | CES:Time | 2.14 | 92.17 | 1.517 | 0.224 |  |
|  | Intensity:Session | 1 | 43 | 0.796 | 0.377 |  |
|  | CES:Session | 1 | 43 | 0.456 | 0.503 |  |
|  | Time:Session | 2.28 | 97.9 | 4.949 | 0.007 | * |
|  | Intensity:Time:Session | 2.28 | 97.9 | 3.07 | 0.044 | * |
|  | CES:Time:Session | 2.28 | 97.9 | 1.397 | 0.252 |  |
| Cortisol | Intensity | 1 | 43 | 0.093 | 0.762 |  |
|  | CES | 1 | 43 | 1.553 | 0.219 |  |
|  | Time | 2.19 | 94.15 | 2.609 | 0.074 |  |
|  | Session | 1 | 43 | 0.082 | 0.777 |  |
|  | Intensity:Time | 2.19 | 94.15 | 0.525 | 0.61 |  |
|  | CES:Time | 2.19 | 94.15 | 2.579 | 0.076 |  |
|  | Intensity:Session | 1 | 43 | 0.21 | 0.649 |  |
|  | CES:Session | 1 | 43 | 0.398 | 0.532 |  |
|  | Time:Session | 2.93 | 126.16 | 1.008 | 0.39 |  |
|  | Intensity:Time:Session | 2.93 | 126.16 | 0.517 | 0.668 |  |
|  | CES:Time:Session | 2.93 | 126.16 | 1.363 | 0.257 |  |
| AA | Intensity | 1 | 42 | 3.197 | 0.081 |  |
|  | CES | 1 | 42 | 1.772 | 0.19 |  |
|  | Time | 3.36 | 141.15 | 5.176 | 0.001 | * |
|  | Session | 1 | 42 | 3.009 | 0.09 |  |
|  | Intensity:Time | 3.36 | 141.15 | 2.143 | 0.09 |  |
|  | CES:Time | 3.36 | 141.15 | 0.943 | 0.429 |  |
|  | Intensity:Session | 1 | 42 | 3.447 | 0.07 |  |
|  | CES:Session | 1 | 42 | 0.395 | 0.533 |  |
|  | Time:Session | 4 | 168 | 0.847 | 0.497 |  |
|  | Intensity:Time:Session | 4 | 168 | 0.981 | 0.419 |  |
|  | CES:Time:Session | 4 | 168 | 1.017 | 0.4 |  |
| Heart rate | Intensity | 1 | 34 | 2.737 | 0.107 |  |
|  | CES | 1 | 34 | 4.851 | 0.035 | * |
|  | Session | 1 | 34 | 0.046 | 0.831 |  |
|  | Intensity:Session | 1 | 34 | 0.000615 | 0.98 |  |
|  | CES:Session | 1 | 34 | 0.036 | 0.851 |  |
| HRV | Intensity | 1 | 34 | 6.838 | 0.013 | * |
|  | CES | 1 | 34 | 0.612 | 0.44 |  |
|  | Session | 1 | 34 | 0.209 | 0.651 |  |
|  | Intensity:Session | 1 | 34 | 0.016 | 0.901 |  |
|  | CES:Session | 1 | 34 | 0.133 | 0.718 |  |
| Respiration | Intensity | 1 | 34 | 1.49 | 0.231 |  |
|  | CES | 1 | 34 | 0.101 | 0.752 |  |
|  | Session | 1 | 34 | 0.417 | 0.523 |  |
|  | Intensity:Session | 1 | 34 | 0.487 | 0.49 |  |
|  | CES:Session | 1 | 34 | 3.55 | 0.068 |  |

**Table S2.** Summary table of the number of participants at each individual-threshold stimulation intensity within both CES groups.

| **CES Intensity (µA)** | **Active (n)** | **Sham (n)** |
| --- | --- | --- |
| 250 | 3 | 8 |
| 300 | 6 | 7 |
| 350 | 4 | 4 |
| 400 | 2 | 1 |
| 450 | 0 | 1 |
| 500 | 6 | 4 |
